# FADS1 promotes the progression of laryngeal squamous cell carcinoma through activating AKT/mTOR signaling

**DOI:** 10.1038/s41419-020-2457-5

**Published:** 2020-04-24

**Authors:** Rui Zhao, Linli Tian, Bo Zhao, Yanan Sun, Jing Cao, Kexin Chen, Fengqing Li, Minghua Li, Desi Shang, Ming Liu

**Affiliations:** 1 Department of Otolaryngology-Head and Neck Surgery, the Second Affiliated Hospital of Harbin Medical University, Harbin, China; 2 College of Life Science, Northeast Forestry University, Harbin, China; 3 Department of Pathology, the Third Affiliated Hospital of Harbin Medical University, Harbin, China; 4 Department of Gerontology, the Second Affiliated Hospital of Harbin Medical University, Harbin, China; 5 College of Bioinformatics Science and Technology, Harbin Medical University, Harbin, China

**Keywords:** Cancer metabolism, Head and neck cancer

## Abstract

Metabolic abnormality is the major feature of laryngeal squamous cell carcinoma (LSCC), however, the underlying mechanism remain largely elusive. Fatty acid desaturase 1 (FADS1), as the key rate-limiting enzyme of polyunsaturated fatty acids (PUFAs), catalyzes dihomo-gamma-linolenic acid (DGLA) to arachidonic acid (AA). In this study, we reported that the expression of FADS1 was upregulated in LSCC, high FADS1 expression was closely associated with the advanced clinical features and poor prognosis of the recurrent LSCC patients after chemotherapy. Liquid chromatograph-mass spectrometry (LC-MS) analysis revealed that FADS1 overexpression induced greater conversion of DGLA to AA, suggesting an increased activity of FADS1. Similarly, the level of prostaglandin E2 (PGE_2_), a downstream metabolite of AA, was also elevated in cancerous laryngeal tissues. Functional assays showed that FADS1 knockdown suppressed the proliferation, migration and invasion of LSCC cells, while FADS1 overexpression had the opposite effects. Bioinformatic analysis based on microarray data found that FADS1 could activate AKT/mTOR signaling. This hypothesis was further validated by both in vivo and in vitro assays. Hence, our data has supported the viewpoint that FADS1 is a potential promoter in LSCC progression, and has laid the foundation for further functional research on the PUFA dietary supplementation interventions targeting FADS1/AKT/mTOR pathway for LSCC prevention and treatment.

## Introduction

Metabolic abnormality is regarded as an important feature of tumors which could help to adjust the microenvironment to meet the requirement of constant-growing tumor cells. Recently, more and more attention has been focused on tumor metabolism to elucidate the pathogenesis of cancer^1,2^, especially on genes related with diet and cancer risk^3^. Polyunsaturated fatty acids (PUFAs), the unsaturated fatty acid containing two or more than two double bonds, can be absorbed from daily diet. They are not only the important components of cell membrane bilayer but also the precursors of many important bioactive molecules in maintaining and modulating biological signaling pathways^4^. LA, which is usually found in cereal oil or red meat products^5–7^, and which is known as the “matrix” of PUFAs, is acquired from diet and cannot be synthesized de novo. LA is converted into ultimate product, AA, through a series of desaturation and elongation reactions. AA-derived eicosanoids include prostaglandins (PGs), leukotrienes (LTs), and thromboxanes (TXs). Epidemiological and biochemical evidence have shown that AA-derived eicosanoids could participate in multiple physiological and pathological processes especially in metabolic syndrome, inflammation and cancer^8–13^. As reported, AA and its downstream metabolite prostaglandin E2 (PGE_2_) could modulate tumor microenvironment, and promote carcinogenesis and angiogenesis by activating PI3K-AKT signaling and mTOR signaling^14–17^. Fatty acid desaturase 1 (FADS1) is the key rate-limiting enzyme of the bioactive metabolites which convert dihomo-gamma-linolenic acid (DGLA) to AA^18^. Previous studies have shown that the expression of FADS1 was dysregulated in many cancers and that knockdown of FADS1 not only inhibited cancer growth and migration but also enhanced the cytotoxicity of chemotherapeutic agents^19–22^. However, the molecular mechanism of FADS1 in laryngeal cancer still remain to be elucidated. Laryngeal squamous cell carcinoma (LSCC) accounts for ~90% of larynx cancer^23^, which is the second most prevalent malignancy occurred in head and neck as well as respiratory tract with high incidence and mortality rate^24–26^. The malignant progression of LSCC initiates from a common type of premalignant lesion called laryngeal severe dysplasia. Poor living habits such as imbalanced diet including dietary fat, smoking and alcohol consumption, are the main risk factors contributed to the incidence of laryngeal cancer^27,28^. Although surgical removal or adjuvant therapy at early stage of LSCC can be often curative, the prognosis of LSCC patients at advanced stages has not been improved yet^29^. Our previous research has discovered that FADS1 and the anabolic anomaly of PUFA were upregulated in LSCC tissues by microarray analysis^30^. Moreover, we also demonstrated that FADS1 variation was significantly associated with laryngeal cancer risk by genome-wide association study (GWAS)^31^. Therefore, in this study, we performed in vitro and in vivo assays, and identified that FADS1, as a main mediator of PUFAs, played an oncogenic role in the progression of laryngeal cancer by activating the AKT/mTOR signaling.

## Materials and methods

### Tissue specimens and cell culture

The paraffin-embedded specimens of 110 LSCC, 30 noncancerous tissues and 30 tissues of laryngeal severe dysplasia were kindly acquired from the Pathology Department in the Second Affiliated Hospital of Harbin Medical University. All 110 LSCC patients were diagnosed by professional pathologist, and were all initially treated by partial or total laryngectomy at the Otorhinolaryngology Department, the Second Affiliated Hospital of Harbin Medical University from February 2008 to October 2012. 74 LSCC patients among them, who underwent tumor recurrence after surgery, had received chemotherapy for further treatment. All 110 patients were followed up for at least 5 years. In addition, 30 paired LSCC and adjacent noncancerous tissues were kindly obtained from patients and were snap frozen in liquid nitrogen within 15 min after excision. All patients provided written informed consent in accordance with the Declaration of Helsinki. The study was approved by the Ethics Committee of The Second Affiliated Hospital of Harbin Medical University. The laryngeal carcinomas cell lines (AMC-HN8, TU212, TU686)^32^ were provided by BeNa Culture Collection (Jiangsu, China). Cells were cultured in DMEM medium (Invitrogen, Carlsbad, CA) containing 10% fetal bovine serum (PAN-Biotech, Adenbach, Germany) and incubated in a humidified 37 °C incubator with 5% CO_2_.

### Lentivirus-mediated plasmid construction and stable cell line preparation

Lentiviral vector encoding the full-length FADS1 sequence (GV492-gcGFP-FADS1) and the Lenti-shRNA vector system (PCDH-GFP) of FADS1 (FADS1-shRNA1/2/3) were purchased from GeneChem (Shanghai, China). GV492-gcGFP vector and shRNA control with a non-targeting sequence were used as control vectors. The sequences of shRNAs were displayed in Table SIA. The lentivirus vector encoding the full-length FADS1 sequence and FADS1-shRNA2 were selected for generating stable overexpressing (OE) and knockdown (KD) cell lines. The cells were incubated in the lentivirus-containing medium supplemented with 4 μg/mL polybrene for 24 h. Seventy-two hours later, cells were then selected by 2.0 μg/mL puromycin for 2 weeks. The stable cell lines were divided into four groups: FADS1-OE and its negative control (NC) group, FADS1-KD and its negative control (Con) group.

### Quantitative real-time polymerase chain reaction (QRT-PCR)

Total RNAs in frozen samples and cell lines were extracted according to the manufacturer’s protocol of TRIzol reagent (Invitrogen, USA) and quantified by the NanoDrop 1000 (NanoDrop Technologies, Rockland, DE, USA). The RNA integrity was evaluated by standard denaturing agarose gel electrophoresis. Total RNAs were treated with RQ1 DNase (Promega). Transcriptor First Strand cDNA Synthesis Kit (Roche Diagnostics) was used for reverse transcription according to the manufacturer’s specification. Real-time PCR was performed in triplicate with SYBR-Green PCR Master Mix (ABI, Foster, CA, USA) on a 7500 Fast Real-Time PCR System (Applied Biosystems, Foster City, CA, USA). The relative expression values of target mRNAs were calculated based on the cycle threshold (CT) and were normalized using β-actin expression as the endogenous control. The primers’ sequences are available in Table SIB.

### Western blot analysis

Proteins in LSCC cell lines (AMC-HN-8, TU212, TU686) and LSCC xenograft tumors in mice were harvested in RIPA buffer and analyzed by western blot, as was described previously^33^. Protein concentrations was quantified by BCA protein quantification kit (KTD3001, Abbkine, CA, USA). Equal amounts of proteins (40 μg) were separated by SDS-PAGE, transferred to polyvinylidene difluoride (PVDF) membrane, and incubated with the following antibodies: anti-FADS1 (1:500 dilution; ab126706; Abcam Biochemicals, UK), anti-AKT1/2/3 (1:500 dilution; ab179463), AKT-Phospho-S473 antibody (1:500 dilution; Proteintech, 66444-1-Ig, CHI, USA), anti-mTOR (1:500 dilution; ab32028), mTOR-phospho-S2448 antibody (1:500 dilution; ab109268), anti-S6K1(1:500 dilution; ab186753), phospho-p70S6 Kinase (Thr421/Ser424) antibody (1:500 dilution; CST, #9204, MA, USA), GAPDH (dilution 1:2000; ab70699). GAPDH expression was used as the loading control.

### ELISA

The stable transfected TU212 cells were seeded at the concentration of 1 × 10^8^ cells per mL. Fifty milligrams of LSCC frozen tissues were homogenized in 1 mL lysis buffer, and the supernatant was collected and the level of PGE_2_ was determined by enzyme-linked immunosorbent assay (ELISA) following the manufacturer’s instruction (Cloud-Clone Corp, Wuhan, China).

### Immunohistochemistry (IHC)

The paraffin specimens were incised by 4-mm thick in human tissues. The immunohistochemistry analysis was performed with anti-FADS1 antigen (1:100 dilution; ab126706; Abcam), AKT-phospho-S473 antibody (1:100 dilution; Proteintech, 66444-1-Ig, CHI, USA), and mTOR-Phospho-S2448 antibody (1:100 dilution; ab109268) as described previously^34^. The assessment of the immunohistochemistry staining was accomplished by two experienced pathologists with unified criteria and single blind method. A score of 4 was used to distinguish between low (≤4) and high (>4) levels of FADS1 gene expression.

### Fatty acid metabolites analysis

Dissected tissue samples were homogenized in H_2_O with acetonitrile/37% hydrochloric acid added to the homogenates. Samples were then hydrolyzed by incubating at 90 °C for 2 h. Fatty acids were extracted with hexane and separated by centrifugation and evaporation to dryness in a vacuum concentrator. Dry extracts were then reconstituted in 50 μL of MeOH/Water (9/1, v/v), and washed by centrifugation to remove insoluble debris. The supernatant was transferred to high-performance liquid chromatography (HPLC) vials and stored at −80 °C prior to liquid chromatograph-mass spectrometer (LC-MS) analysis. Samples were separated on a Poroshell 120 EC-C18 column (2.7 μm, 3.0 * 150 mm, Agilent), using mobile phase A containing water mixed with 0.1% formic acid, and mobile phase BACN with 0.1% formic acid. MS analysis was carried out on Agilent 6460 MS/MS in negative ion mode. Peaks of the fatty acids were identified by comparing retention time with reference standards obtained from Nu-chek Prep (Elysian, MN, USA) and Sigma-Aldrich (MO, USA). Data acquisition and analysis were performed using QuantAnalysis (Agilent, Santa Clara, CA, USA).

### Cell proliferation assay

Cell viability was assessed with the cell counting kit 8 (CCK-8, Sigma-Aldrich, MO, US) and colony formation assay according to the manufacturer’s instruction (BestBio Molecular Technologies, ShangHai, China). For CCK-8 assay, transfected LSCC cell lines were incubated with 10 μL CCK-8 each well in a 96-well plate. Absorbance was quantified at a wavelength of 450 nm with a microplate reader (BioRad, Richmond, CA, USA). For colony formation assay, cells were seeded into six-well plates (800 cells/well) and stained with crystal violet after 14 days incubation. The number of colonies containing more than 50 cells was determined by cell counting.

### Flow cytometry analysis

Annexin V-PE/7-AAD Apoptosis Detection kit (KeyGen Biotech, NanJing, China) was used to assess the apoptosis of cells. Cells were resuspended with 500 μL binding buffer at a concentration of 1 × 10^5^ cells/mL and incubated with 5 μL Annexin V-APC at room temperature in the dark for 10–15 min. Fluorescence of cells was detected by flow cytometry (FACSCalibur; Becton Dickinson Immunocytometry Systems, San Jose, CA, USA) within 1 h. For cell cycle assay, the cells were collected, fixed with 75% cold ethanol after washing twice by phosphate-buffered saline (PBS), and stained with ethidium bromide for 15 min at 37 °C. The DNA content and cell cycle distribution of cells were analyzed by flow cytometry and ModFit LT (Mac V3, Verity Software House, USA).

### Transwell assays

The migration and invasion assays were performed using BD Falcon Cell Culture Inserts (BD Biosciences, San Jose, CA). For migration assay, cultured cells at the concentration of 1 × 10^4^ cells/mL were appended to the upper compartment of a transwell chamber (24-well, 8-μm pores) and were incubated for 24 h at 37 °C. After 24 h in culture, the migrated cells attached to the lower membrane were stained with 0.1% crystal violet in PBS for 20 min and counted for five individual fields with ×200 magnification. For invasion assay, transwell filters were first coated with matrigel (BD Biosciences), and similar subsequent experimental procedure to the migration assay was performed.

### Wound-healing assay

The stable cells were seeded in 6-well plates and cultured to about 80% confluence. The monolayer cells were scratched using 1 mL pipette tip. Migrative ability was evaluated by measuring the scratch area which was not covered by cells. The representative wounds 24 h after scratching were captured by the microscope (×100 magnification).

### Animal experiments

Five-week-old male BALB/c nude mice were purchased from Vital River Laboratories (Beijing, China). The transfected stable cell lines (FADS1-OE and NC groups, FADS1-KD and Con groups) were harvested aseptically, and 100 μL suspension at the concentration of 1 × 10^8^ cells was injected subcutaneously into the neck area of the nude mice to establish subcutaneous xenograft. All mice were randomly divided into different groups (*n* = 6/group). Celecoxib, a COX2-selective inhibitor was administered in the food at 250 mg/kg/day p.o.^35^ when FADS1-OE tumors reached a mean volume of 0.1 mm^3^. Mice were euthanized at 5 weeks after tumor transplantation for tumor evaluation. Tumor size was measured with digital caliper every 3–5 days and tumor volume was determined as the following formula: length × width^2^ × 0.5. All data of animal experiments were acquired blindly. Animal experiments were approved by the Animal Ethics Committee of The Second Affiliated Hospital of Harbin Medical University and performed according to the National Institute of Health Guide for the Care and Use of Laboratory Animals.

### Microarray analysis

The stable FADS1-OE and NC cells were used for transcriptome profile by the Agilent One-Color Gene Expression Microarray (KangChen Bio-Tech Inc.). Total RNAs were extracted from the cells transfected with FADS1 overexpressed lentivirus vector and empty vector using TRIzol reagent (Invitrogen). Sample labeling and array hybridization were performed according to the protocol (Agilent Technology). Raw data were log2-transformed and normalized by quantile normalization method. Differentially expressed genes (DEGs) were further identified through the standard of fold change (FC) ≥ 1.5 and *P* value < 0.05 (FC ≥ 1.5, *P* < 0.05). The Gene Ontology (GO) biological process and Kyoto Encyclopedia of Genes and Genomes (KEGG) were used to perform functional annotation enrichment of the DEGs and were ranked by *P* value. *P* value of < 0.05 was regarded as significant difference. The microarray original data are available in Supplementary dataset I.

### Integration of protein–protein interaction (PPI) network and module analysis

We used the online Search Tool for the Retrieval of Interacting Genes (STRING) database, which is designed to evaluate the protein–protein interaction (PPI) information. By mapping the differentially expressed genes (DEGs) to STRING, we extracted the interactions between DEGs and their first neighbors. Only experimentally validated interactions with a combined score >0.9 were selected as significant results. PPI networks were constructed by the Cytoscape software^36^. The modules of PPI network were identified using the plug-in Molecular Complex Detection (MCODE).

### TCGA database analysis

A web-based tool based on TCGA and GTEx data, Gene Expression Profiling Interactive Analysis (GEPIA)^37^, was used to compare the expression of FADS1 in HNSC with that in normal tissues. Meanwhile, FADS1 expression in different cancer stages and the expression correlation between FADS1 and AKT, mTOR, S6K1 were also calculated with GEPIA, which could provide key interactive and customized functions including differential expression analysis, profiling plotting, correlation analysis, patient survival analysis, similar gene detection and dimensionality reduction analysis.

### Statistical analysis

Data were presented as mean ± standard errors (SEM). The Student’s *t*-test was used for the comparison of measurable variants of two groups. The relationship between FADS1 expression and the clinicopathological parameters was evaluated by Chi-square test. The significant difference of overall survival (OS) between groups was compared using the Kaplan–Meier method assessed by the log-rank test. Statistical analysis was performed by the GraphPad Prism software package (v. 4.02; San Diego, CA) or SPSS 16.0 software (SPSS, Chicago, IL, USA). All the experiments were performed in triplicates. A criterion of *P* < 0.05 was regarded as statistically significant for all comparisons.

## Results

### FADS1 is upregulated in LSCC tissues

**Fig. 1:**
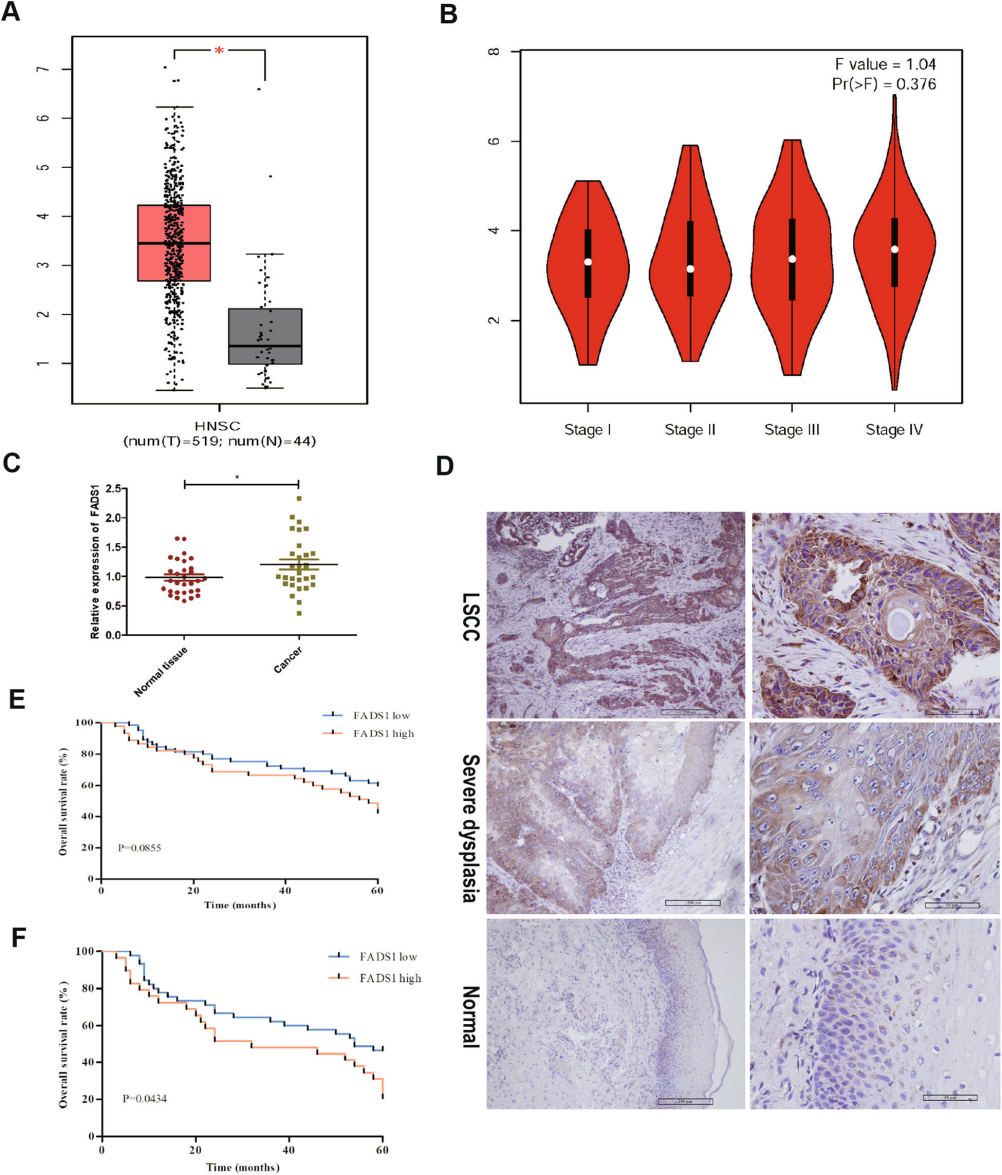
FADS1 is upregulated in LSCC tissues and predicts poor survival. **a** Box plots showed mRNA levels of FADS1 in HNSC tissues and noncancerous tissues in TCGA datasets from GEPIA. **b** Box plots showed that expression levels of FADS1 were gradually increased with T stage progression. **c** QRT-PCR analysis of FADS1 mRNA in 30 paired LSCC tumor tissues. *P < 0.05, two-tail paired *t*-test. **d** Immunohistochemistry staining of FADS1 in LSCC tissues, laryngeal severe dysplasia tissues and normal laryngeal tissues under ×100 and ×400 magnification respectively. **e** Kaplan–Meier overall survival analysis stratified by FADS1 expression in patients with LSCC. **f** Kaplan–Meier overall survival analysis stratified by FADS1 expression in patients with LSCC recurrence and chemotherapy.

The expression and clinical significance of FADS1 were assessed by GEPIA based on high-throughput RNA-sequencing data of HNSC cohort of the TCGA database. Results showed that the expression level of FADS1 was significantly upregulated in HNSC samples compared with that in nonmalignant samples and was gradually increased with clinical stage progression (Fig. 1a, b). We then verified the FADS1 mRNA expression and found that the mRNA level of FADS1 was enhanced in 30 LSCC cancer tissues than that in paired nonneoplastic tissues by qRT-PCR (Fig. 1c). In addition, paraffin specimens from 110 LSCC, 30 noncancerous and 30 laryngeal severe atypical hyperplastic tissues were used to perform IHC detection. Results indicated that the positive rate of FADS1 in LSCC tissues, laryngeal severe atypical hyperplastic and noncancerous tissues were 100%, 96.7%, and 73.3%, respectively. Moreover, high level of FADS1 expression (scoreå 4) was detected in 45 LSCC cases (40.9%), seven laryngeal severe dysplasia cases (23.3%) and three noncancerous cases (10%). Accordingly, the average expression level of FADS1 in laryngeal severe atypical hyperplastic group was significantly higher than that in noncancerous group and lower than that in LSCC group (*P* < 0.05) (Fig. 1d). We further evaluated the relationship between FADS1 expression level and each clinicopathological factor in LSCC paraffin specimens. Data demonstrated that the expression of FADS1 was statistically associated with T classification and clinical stage. Tumors of Grade T3 to T4 or advanced clinical stages expressed higher levels of FADS1 (Table SII, *P* < 0.05). Although Kaplan–Meier analysis did not exhibit significant association between FADS1 expression and the overall survival in 110 LSCC patients who underwent laryngectomy (Fig. 1e), the overall survival with high FADS1 expression was significantly lower than that with low FADS1 expression in those 74 patients who underwent LSCC recurrence and received chemotherapy (Fig. 1f). These observations suggested the involvement of FADS1 in LSCC progression.

### The bioactivity of FADS1 is enhanced in LSCC

**Fig. 2:**
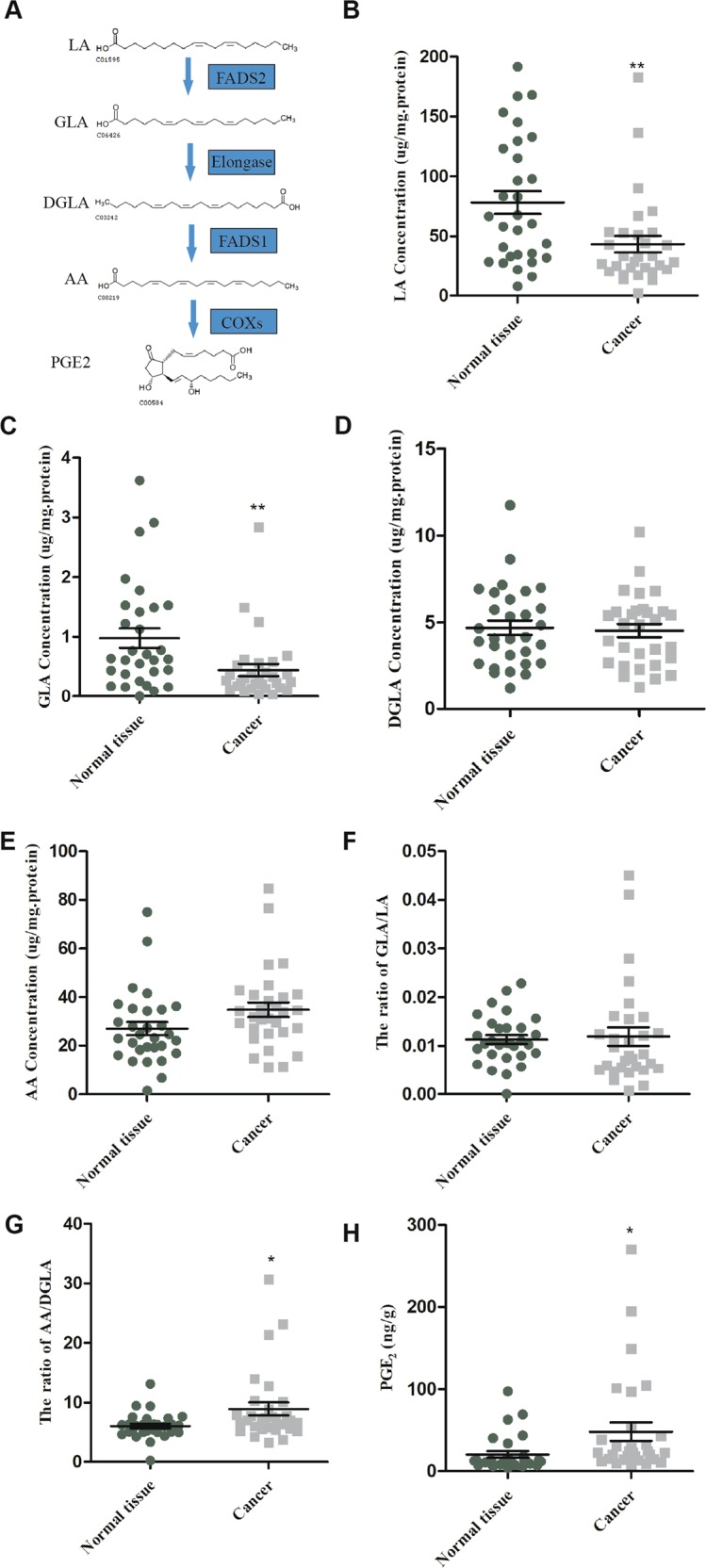
Fatty acid profile of the FADS1 pathway in LSCC tissues. The FADS1 pathway of LA to PGE_2_ (**a**). The fatty acids expression of FADS1 pathway in tumor and non-tumor larynx tissues, including LA (**b**), GLA (**c**), DGLA (**d**), and AA (**e**). The ratio of GLA/LA (**f**) and AA/DGLA (**g**). The level of PGE_2_ (**h**). *P < 0.05, **P < 0.01 by Student’s *t*-test.

FADS1 functioned as a rate-limiting enzyme converting DGLA to AA, and its activity could be estimated by the ratio of AA to DGLA (Fig. 2a). To determine whether FADS1 activity is dysregulated in LSCC, we analyzed the key PUFA metabolomic productions (LA, GLA, DGLA, AA and PGE_2_) in 30 paired cancerous and noncancerous tissues. Based on HPLC-MS results, we detected the content of these key PUFAs (LA, GLA, DGLA, AA) in 30 paired LSCC tissues. The standard curve of each lipid metabolite in tissues was shown in Supplementary Fig. 1. We then measured the PGE_2_ content by ELISA detection. Data showed that there was a lower concentration of LA and GLA in LSCC tissues compared with the corresponding noncancerous tissues (*P* < 0.05). In contrast, both the ratio of AA to DGLA and the level of PGE_2_ were significantly higher in cancerous than noncancerous tissues (*P* < 0.05) (Fig. 2b–h), which indicated that the activity of FADS1 was significantly enhanced in LSCC tissues.

We manipulated FADS1 expression in TU212 cells by lentivirus transfection to observe FADS1 bioactivity, after assessing FADS1 levels in laryngeal carcinoma cell lines (AMC-HN-8, TU212, TU686) (Supplementary Fig. 2A–C). Compared with negative control, FADS1 expression was most notably decreased by Lenti-shRNA2 and moderately decreased by other two shRNAs as was shown in Supplementary Fig. 2D, E. Therefore, the lentivirus vector encoding GV492-gcGFP-FADS1 and FADS1-shRNA2 were chosen for the construction of stable cell clones. FADS1 expression in TU212 stable cells was confirmed by qRT-PCR and western blot (Supplementary Fig. 2F, G). We calculated the content of the relevant lipid metabolites (LA, GLA, DGLA, AA) by HPLC-MS (Supplementary Fig. 2H–K). The ratio of AA to DGLA was significantly higher when FADS1 was upregulated and vice versa (Supplementary Fig. 2L). ELISA data indicated that the level of PGE_2_ was apparently higher in FADS1-OE cells and lower in FADS1-KD cells compared with the control vectors (Supplementary Fig. 2M). The standard curve of each lipid metabolite in stable transfected TU212 cells was shown in Supplementary Fig. 3. Collectively, these data showed that the activity of FADS1 might be in significant positive correlation with the expression level of FADS1 in vitro.

### FADS1 promotes LSCC cell proliferation, migration, and invasion

**Fig. 3:**
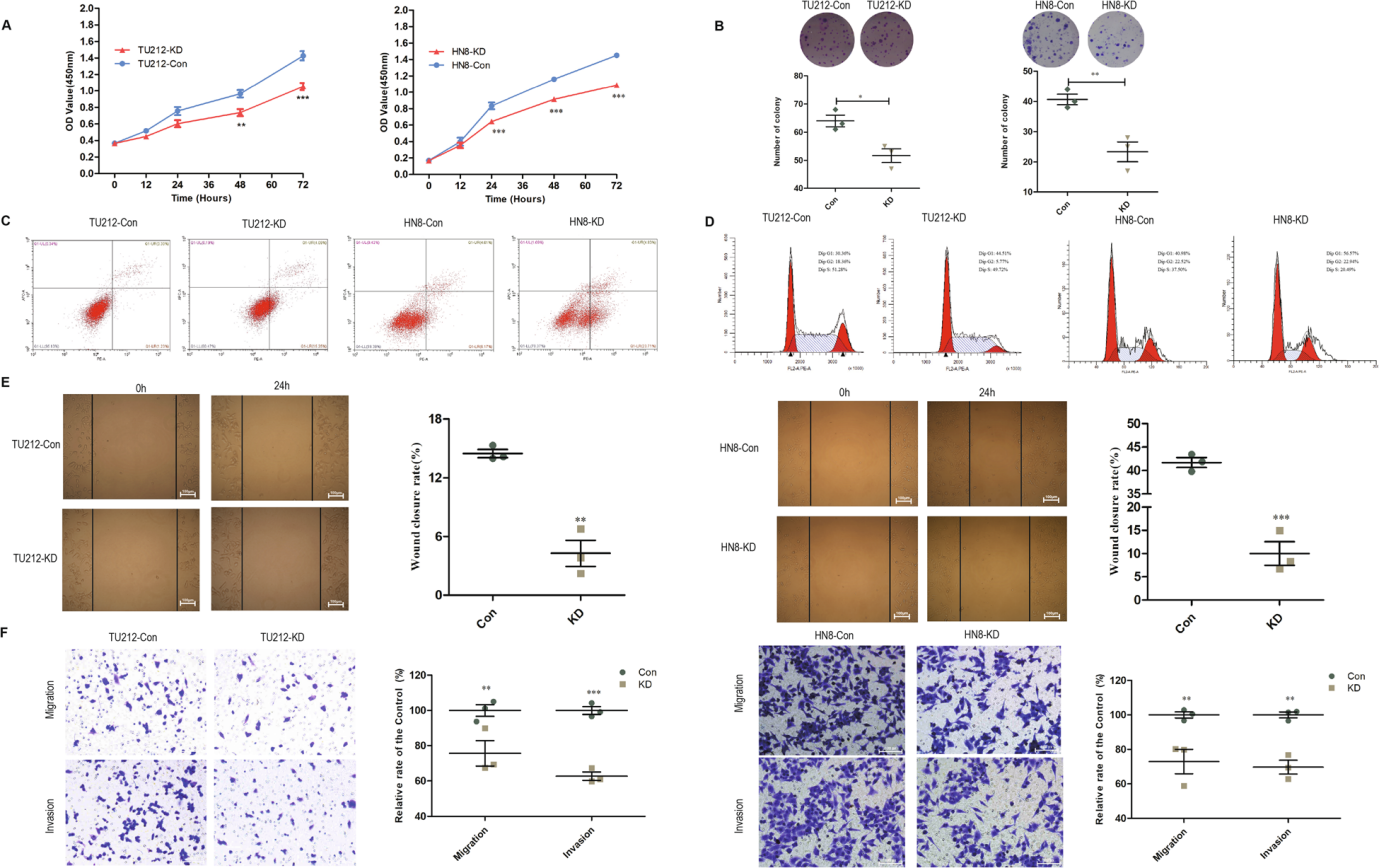
The effect of FADS1 depletion on the proliferation, migration and invasion of laryngeal cancer cell lines (TU212 and HN8). **a** CCK-8 assay was conducted to determine the proliferative ability of the indicated LSCC cells (Con, KD). **b** Colony formation assay was conducted in soft agar and an average colony number in multiple fields was counted in FADS1-KD and Con cells. **c** Flow cytometry analysis of cell apoptosis of the indicated laryngeal cancer cells (Con, KD) using propidium iodide/annexin V double staining. **d** Representative images of the cell cycle analysis of the indicated laryngeal cancer cells (Con, KD). **e** The representative images of the wound-healing assay of the indicated laryngeal cancer cells (Con, KD). Migration was monitored by light microscopy at 0 h and 24 h with ×100 magnification. **f** Transwell assay was conducted to detect the migratory and invasive ability of the indicated laryngeal cancer cells (Con, KD) with ×200 magnification. Quantitative analysis was shown on the right side of each representative figure. *P < 0.05, **P < 0.01, ***P < 0.001 by Student’s *t*-test. All experiments were performed in triplicates and data are presented as mean ± SEM.

To investigate the influence of FADS1 on the malignant growth and metastasis potential, we transfected GV492-gcGFP-FADS1 and FADS1-shRNA2 vector into both TU212 and AMC-HN8 cells. FADS1 expression was confirmed by qRT-PCR and western blot (Supplementary Figs. 2F, G; 4A, B). CCK-8 and colony formation assay were performed to assess the cell proliferation ability. Compared with Con group, FADS1 depletion markedly inhibited tumor cell growth (Fig. 3a). Similarly, the number of cell colonies was apparently reduced in FADS1-KD group than that in Con group (Fig. 3b). We further used flow cytometry analysis to investigate cell apoptosis and cell cycle of each group. Compared with the Con group, the percentage of apoptotic cells was conspicuously higher in FADS1-KD group (Fig. 3c). Moreover, as G1 cell cycle arrest is one of the main biomarkers of cellular senescence, we found that the G1 phase was markedly prolonged in FADS1-KD group (Fig. 3d). Thus, our results demonstrated that FADS1 downregulation could suppress the viability of laryngeal cancer cells by reducing cell proliferation, promoting cell apoptosis and senescence. In addition, wound-healing and transwell assay were performed to observe the effect of FADS1 on the ability of cell motility. In the wound-healing assay, we found that FADS1 knockdown remarkably inhibited cell migration (Fig. 3e). Meanwhile, the transwell assay showed that FADS1-KD cells migrated and invaded more slowly than control cells (Fig. 3f). These results indicated that FADS1 knockdown could significantly reduce the proliferative and metastatic capabilities of laryngeal cancer cells.

**Fig. 4:**
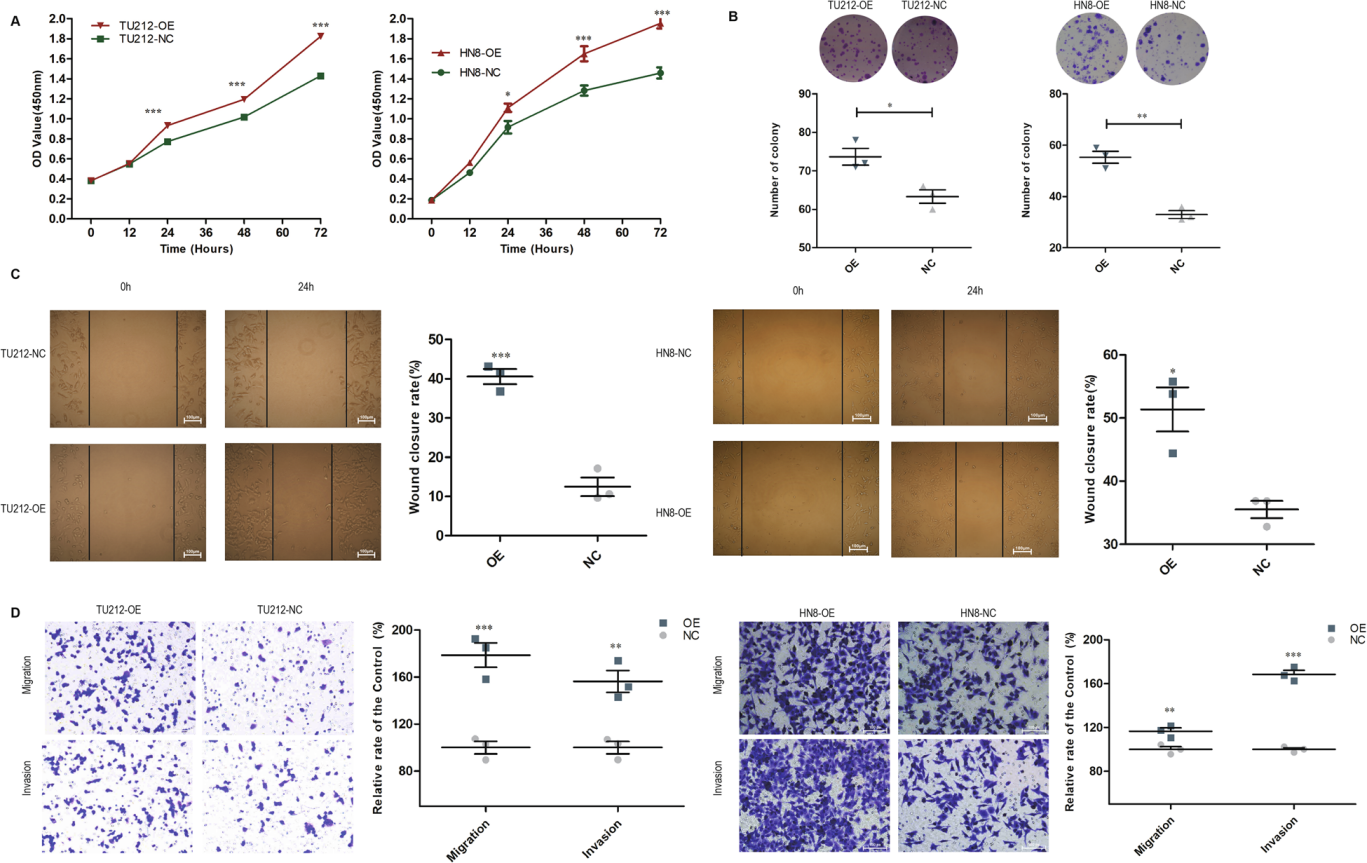
The effect of FADS1 overexpression on proliferation, migration and invasion of laryngeal cancer cell lines (TU212 and HN8). **a** Growth curves of FADS1-OE and NC cells were determined by CCK-8 assay. **b** Colony formation assay was conducted and an average colony number in multiple fields was counted in FADS1-OE and NC cells. **c** The representative figure of the wound-healing assay of the indicated laryngeal cancer cells (OE, NC). Migration was monitored by light microscopy at 0 h and 24 h with ×100 magnification. **d** Transwell assay was conducted to detect the migratory and invasive ability of the indicated laryngeal cancer cells (OE, NC) with ×200 magnification. Each representative image was shown with quantitative analysis on the right side. *P < 0.05, **P < 0.01, ***P < 0.001 by Student’s *t*-test. All experiments were performed in triplicates and all data are represented as mean ± SEM.

Accordingly, the growth curve and colony formation assays indicated that FADS1-OE cells proliferated significantly faster than NC cells (Fig. 4a, b). FADS1 overexpression significantly promoted the migration and invasion of TU212 and AMC-HN8 cells by the wound-healing and transwell assay (Fig. 4c, d).

### Microarray analysis reveals FADS1-mediated dysregulated mRNAs profile in laryngeal cancer cells

**Fig. 5:**
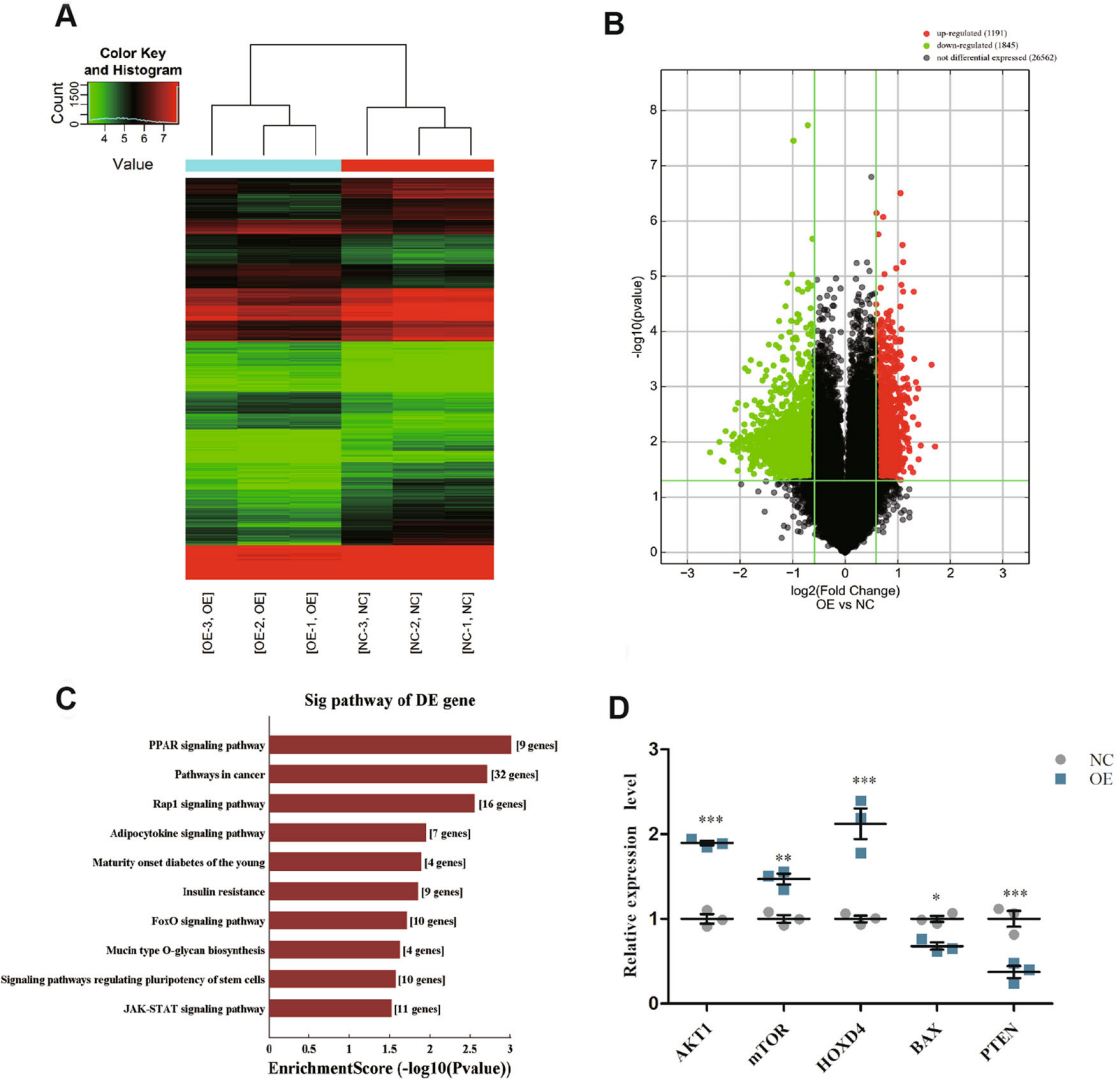
Microarray analysis of FADS1-OE cells versus FADS1-NC cells. Hierarchical clustering (**a**) and volcano plots (**b**) for the expression profiles of mRNAs. **c** The top 10 significant terms by KEGG pathway analysis for differential expressed mRNAs in the microarray. **d** Verification of the differentially expressed transcripts by qRT-PCR in 30 paired LSCC tissues. *P < 0.05, **P < 0.01, ***P < 0.001, two-tail paired *t*-test.

To elucidate the underlying functional mechanism of FADS1 in LSCC tumorigenesis, we attempted to characterize the aberrant transcriptome profile in FADS1-OE TU212 cells compared with NC cells by gene expression microarray. We found that FADS1 could potentially upregulate 1191 genes and downregulate 1845 genes at the cutoff of FC ≥ 1.5, *P* < 0.05. We performed hierarchical clustering analysis and drew volcano plots to provide an overview of the expression profiling characteristics based on all the microarray data (Fig. 5a, b). Functional annotation revealed that the upregulated mRNAs were enriched in PPAR signaling, pathways in cancer and etc. (Fig. 5c). The details of annotated genes were displayed in Table SIII. We then evaluated five DEGs including two upregulated and three downregulated DEGs of the microarray data, and confirmed their expression consistency in cells by qRT-PCR (Fig. 5d).

### FADS1 attenuates AKT/mTOR pathway both in vivo and in vitro

**Fig. 6:**
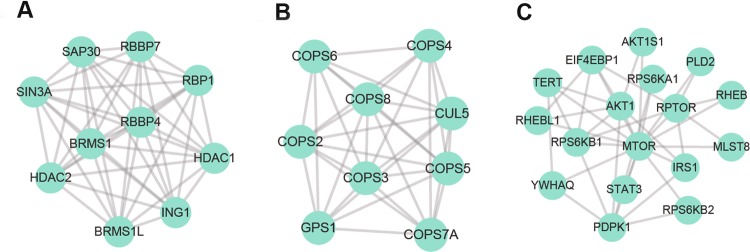
The top three significant modules in PPI network. **a** Module 1, **b** module 2, and **c** module 3.

**Fig. 7:**
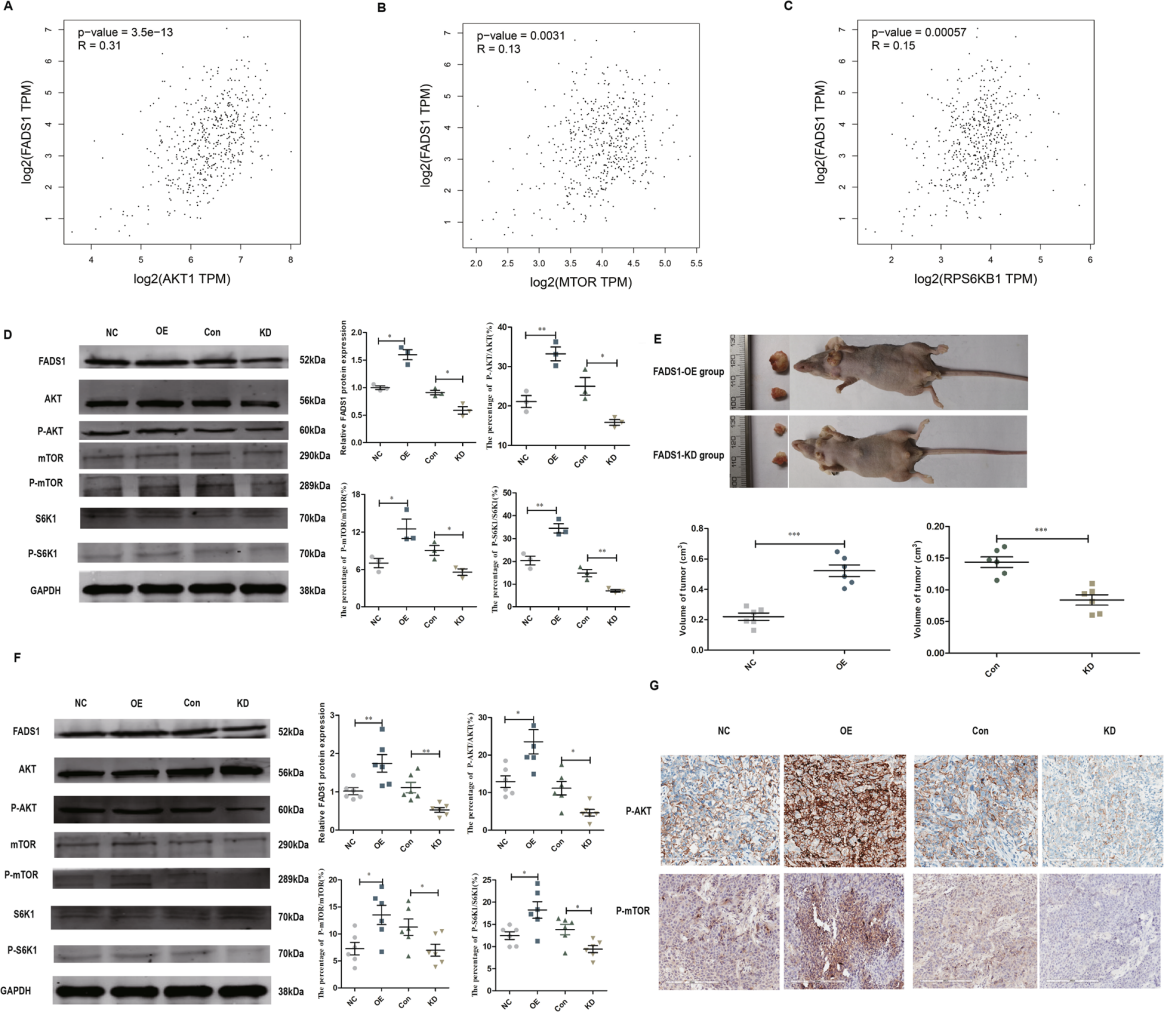
AKT-mTOR signaling is involved in FADS1-mediated action. The expression relationship between FADS1 and AKT1 (**a**), mTOR (**b**), S6K1 (**c**) in HSNC in published database from GEPIA. **d** AKT, p-AKT and its downstream targets (mTOR, S6K1) were evaluated by western blot in the indicated laryngeal cancer cells (NC, OE, Con, KD). **e** The representative figure of xenograft tumors of FADS1-OE group and FADS1-KD group (*n* = 6/group). The tumors formed by FADS1-OE cells were obviously larger compared with FADS1-NC cells while the tumors formed by FADS1-KD cells were remarkably smaller than by FADS1-Con cells. **f** The expression of FADS1, AKT, p-AKT, mTOR, p-mTOR, S6K1, and p-S6K1 in the xenograft tumors were examined by western blot. Each representative image was shown with quantitative analysis on the right side. **g** The expression of p-AKT and p-mTOR were examined by IHC staining in the xenograft tumor tissues. *P < 0.05, **P < 0.01, ***P < 0.001 by Student’s *t*-test. All data are represented as mean ± SEM.

**Fig. 8:**
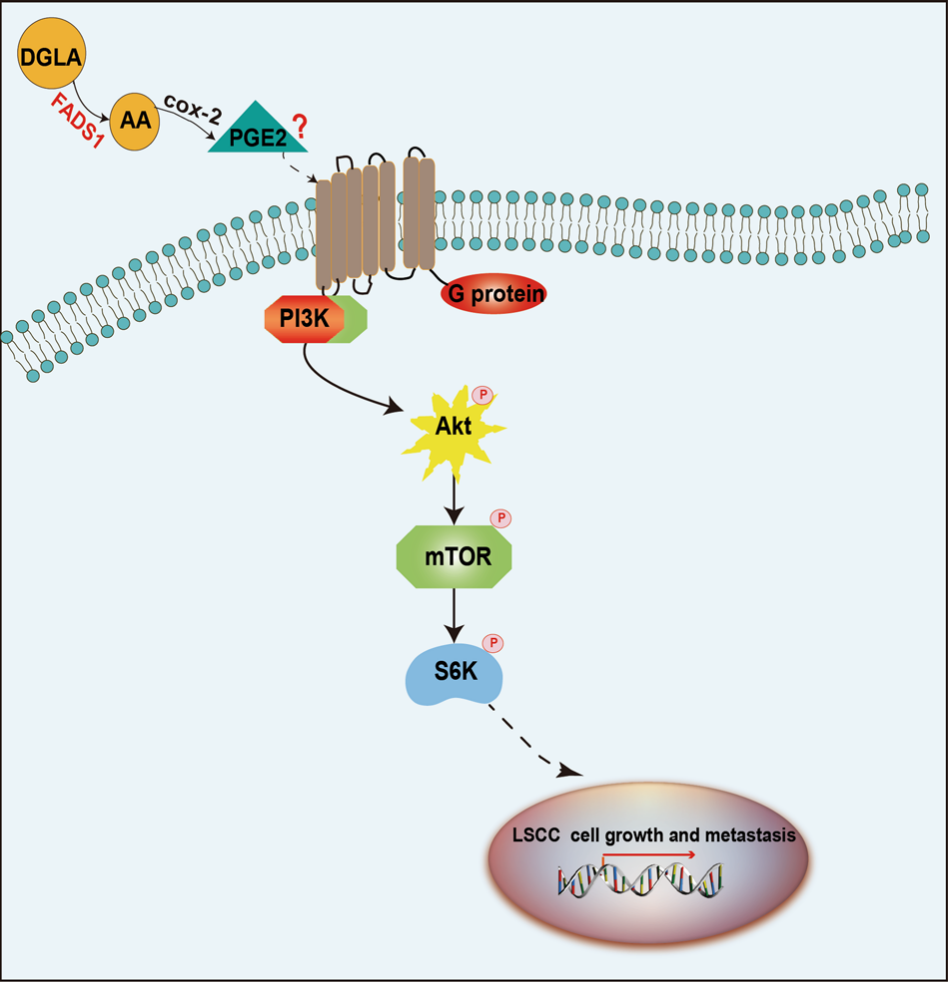
Schematic figure showing the functional mechanism pathway of FADS1. FADS1 promotes the cell proliferation and metastasis of LSCC by increasing the phosphorylation of AKT, mTOR and S6K1.

To acquire a better understanding of downstream pathways of FADS1, we used the above DEGs to construct PPI network (Supplementary Fig. 5) and identified the modules in this network by MCODE plug-in as candidates based on the information in the STRING database. The information of the PPI network was provided in Supplementary dataset II. The top three significant modules were displayed in Fig. 6a–c, which were found to be highly related to AKT/mTOR signaling. Besides, we simultaneously evaluated the expression correlation between FADS1 and AKT, mTOR, RPS6KB1(S6K1) in HNSC by GEPIA, and data showed that they are all positively correlated (*P* < 0.01) (Fig. 7a–c). For further validation, we examined the activation of AKT, mTOR, S6K1 in the transfected stable cell lines. Compared with NC cells, FADS1 overexpression could induce the phosphorylation of AKT, mTOR, and S6K1. However, the phosphorylation of AKT, mTOR, and S6K1 were significantly reduced when FADS1 was knocked down (Fig. 7d). To determine whether the above results acquired in vitro could also be recapitulated in vivo, we established FADS1 xenograft model in nude mice by injecting with the stable transfected cells with FADS1 overexpression or silencing (*n* = 6/group). As illustrated in Fig. 7e, when FADS1 was overexpressed, the average xenograft tumor volume was increased about twofold compared with NC group. Conversely, the average tumor volume with FADS1 silencing decreased by approximately one third compared with control group. Results showed that FADS1 depletion repressed tumor growth and tumor weight of xenograft tumors. Typically, an earlier xenograft formation was observed in FADS1-OE group (14.6 ± 1.85 days) in comparison to NC group (19.2 ± 2.04 days). On the other hand, the appearance of the xenograft was lagged behind in FADS1-KD group (24.2 ± 1.93 days) compared with control group (19.4 ± 2.94 days). The tumor volume growth curves of nude mice over time were shown in Supplementary Fig. 6. The expression of FADS1, AKT, mTOR, and S6K1 in tumor tissues was also determined by western blot and IHC, which was basically in accordance with the results in vitro that FADS1 increase the phosphorylation level of AKT, mTOR, and S6K1 (Fig. 7f, g). In addition, we downregulated the expression of PGE_2_ by celecoxib treatment in FADS1-OE xenograft mouse models and found celecoxib could inhibit the FADS1-driven tumor growth (Supplementary Fig. 7). Thus, we demonstrated that FADS1 could promote the LSCC progression by activating the AKT-mTOR pathway and delineated the underlying functional mechanism of FADS1 in Fig. 8.

## Discussion

Healthy diet or not, has been regarded as a potentially important determinant of cancer risk especially for the upper aerodigestive tract (UADT) cancers (oral cavity, pharynx, larynx, and esophagus)^38,39^. The consumption of dietary lipids has been reported to be associated with cancer risk^3,40^. PUFAs are the main component of cell membrane phospholipids bilayer which play pivotal roles in maintaining membrane structure, energy provision, and cell signaling^41,42^. Of note, studies have indicated that PUFAs may be critical for cancer incidence^43–45^. PUFAs and its derived metabolites, coordinating signal transduction and microenvironment to accelerate tumor growth and spread. However, the exploration of new treatment from the perspective of PUFAs was often ignored until abnormal expression of PUFAs metabolites was found in a variety of tumors^46–48^, suggesting potential cancer therapeutic targets in the PUFAs metabolic pathway. Linoleic acid (LA), as a “matrix” of PUFAs, finally converts into the 20-carbon product-arachidonic acid (AA) through a series of oxidative desaturation and elongation reactions. AA-derived eicosanoids (PGs, TXs, and LTs) are demonstrated to affect tumor development by altering tumor microenvironment in many types of cancer^49–54^. FADS1, as the rate-limiting enzyme of PUFA synthesis, catalyzing DGLA to AA, was reported to be frequently dysregulated in cancers. FADS1 knockdown not only inhibited cancer growth and migration but also enhanced the cytotoxicity of chemotherapy drug^19–21,55^.

Interestingly, our previous research has identified the FADS1 overexpression and the anabolic anomaly of PUFA in LSCC tissues by microarray and functional annotation analysis. We also found that FADS1 variation was significantly associated with laryngeal cancer risk by genome-wide association study (GWAS), combined with GEPIA analysis and experimental data. We also revealed that FADS1 was upregulated in LSCC tissues and had significant correlation with TNM stage. The Kaplan–Meier analysis illustrated that high levels of FADS1 correlated with poor survival of LSCC patients after LSCC recurrence and chemotherapy. Previously, FADS1 knockdown was reported to induce cell apoptosis and enhance the cytotoxicity of 5-fluorouracil (5-FU), which suggested that FADS1 may be an essential factor to predict chemotherapeutic efficacy in LSCC. Thus, we proposed the hypothesis that FADS1, as a potential therapeutic target of PUFAs, might play an important role in LSCC pathogenesis. We then detected FADS1-related key PUFA metabolic productions (LA, GLA, DGLA, AA) in LSCC samples and cells validating that FADS1 activity (AA/DGLA) was enhanced when FADS1 was upregulated. Further, in vivo and in vitro results demonstrated that FADS1 could regulate cell proliferation, migration, invasion in TU212 and AMC-HN8 cell lines. Our data highlighted the importance of FADS1 in promoting LSCC tumorigenicity. However, it still remains to be elucidated about what is the underlying mechanism for FADS1 in LSCC progression.

To elucidate the mechanism of FADS1 in LSCC, we evaluated the dysregulated transcriptome profile in FADS1-OE TU212 cells compared with control cells by genomic microarray. We found that DEGs including AKT, mTOR, S6K1 were highly associated with AKT/mTOR signaling. It is well-known that AKT/mTOR signaling play an important role in cell growth and proliferation^56,57^. The expression level of FADS1 and AKT, mTOR, S6K1 in HNSC was calculated by GEPIA to be significantly positively correlated. Our in vitro and in vivo data further confirmed that FADS1 could modulate these genes by increasing the phosphorylation of AKT, mTOR and S6K1.

PGE_2_, a downstream metabolite of AA, is abundant in various kinds of human malignancies including head and neck cancer and predict poor prognosis^58^. PGE_2_ functions by binding to EP receptors (EP1, EP2, EP3, and EP4), which belong to the members of G double protein receptors. Therefore, PGE_2_ activates its downstream signaling pathways through G protein coupling mechanism^59^. PGE_2_ and its receptors have important role in cancer development, proliferation, apoptosis, angiogenesis, immunosuppression, tumor invasion, and metastases^60^. In addition, previous studies^15–17^ indicated that PGE_2_, acting through EP2/4 receptors, could increase AKT, p70S6K, and S6 phosphorylation and activate PI3K/AKT/mTOR pathway. In turn, rapamycin completely blocked the effects of PGE_2_ on phosphorylation of p70S6K and S6. Since PGE_2_ could act as a trigger of AKT-mTOR signaling, we measured the PGE_2_ content in LSCC tissues and cells, and found that FADS1 produces high levels of PGE_2_. Meanwhile, PGE_2_ was downregulated by celecoxib in FADS1-OE xenograft mice model. The antitumor activity of celecoxib was appeared to reduce the average volume of the xenograft tumors driven by FADS1. Consistent with our finding, the tumor growth was reported to be PGE_2_ dependent in HNSCC xenograft models^35^. Celecoxib could block the production of PGE_2_ and inhibit the growth of HNSCC cell lines^61^. Thus, we deduced that PGE_2_ might be a bridge to connect FADS1 and AKT/mTOR signaling pathway. However, further studies are still needed to validate this hypothesis.

In conclusion, we found that FADS1 expression and its activity were upregulated in LSCC samples. PGE_2_ was positively correlated with FADS1 activity. FADS1 overexpression could promote LSCC tumor growth and metastasis both in vitro and in vivo by activating AKT/mTOR signaling pathway. Our research elucidated a novel mechanism of FADS1-mediated laryngeal carcinogenesis and provided new perspectives for the therapeutic targets, including FADS1/AKT/mTOR axis or PUFA-nutritional interventions in the clinical prevention and treatment of LSCC. Finally, future work should consider the development of potential applications about non-pharmaceutical intervention methods to address FADS1 activity, focusing on PUFA rich products or pharmaceutical FADS1 inhibition in LSCC prevention.

## Supplementary information

Supplementary Figure Legends

Supplementary Fig. 1

Supplementary Fig. 2

Supplementary Fig. 3

Supplementary Fig. 4

Supplementary Fig. 5

Supplementary Fig. 6

Supplementary Fig. 7

Table SI

Table SII

Table SIII

Supplementary datasetI

Supplementary dataset II
