# Supplementary material for: FADS1 promotes the progression of laryngeal squamous cell carcinoma through activating AKT/mTOR signaling
Source: Cell Death Dis. 2020 Apr 24;11(4):272. doi: 10.1038/s41419-020-2457-5 (PMC7181692; doi:10.1038/s41419-020-2457-5)
Supplement: Supplementary file 1 — Supplementary Figure Legends [file 41419_2020_2457_MOESM1_ESM.doc]

**Supplementary Figure Legends**

**Supplementary Fig. 1** The standard curve of PUFAs: LA (**A**), GLA (**B**), DGLA (**C**), AA (**D**) in LSCC tissues.

**Supplementary Fig. 2** Fatty acid profile of the FADS1 pathway in the indicated LSCC laryngeal carcinomas cells. **A** QRT- PCR analysis of FADS1 mRNA in three laryngeal carcinomas cell lines (TU686, TU212, AMC-HN8). **B** Western blot analysis of FADS1 expression in three laryngeal carcinomas cell lines (TU686, TU212, AMC-HN8). **C** Images of TU212 cells 72 hs after transduction with negative control (Con and NC) lentivirus in light microscope and fluorescence microscope (200×). FADS1 expression level in TU212 cells by lentiviral delivery of FADS1 shRNA1-3 compared with the control detected by qRT-PCR (**D**) and western blot (**E**). Relative expression level of FADS1 in stable TU212 cells (NC, OE, Con, KD) was detected by qRT-PCR (**F**) and western blot(**G**). The expression of fatty acids of FADS1 pathway in laryngeal cancer cell lines (NC, OE, Con, KD), including LA (**H**), GLA (**I**), DGLA (**J**), AA (**K**). the ratio of AA/DGLA (**L**) and the level of PGE_2_ (**M**). *P<0.05, **P < 0.01 by Student’s t-test. All the data are represented as mean ± SEM.

**Supplementary Fig. 3** The standard curve of LA (**A**), GLA (**B**),DGLA (**C**), AA (**D**) in laryngeal cancer cells (OE, NC, KD, Con).

**Supplementary Fig. 4** Relative expression level of FADS1 in stable HN8 cells (NC, OE, Con, KD) was detected by qRT-PCR (**A**) and western blot (**B**).

**Supplementary Fig. 5** PPI network of differentially expressed genes (DEGs) in the microarray.

**Supplementary Fig. 6** The tumor volume growth curves of nude mice over time.

**Supplementary Fig. 7** The FADS1-OE mice xenograft models after celecoxib treatment. **A** the PGE_2_ expression level was downregulated in the FADS1-OE xenograft tumor tissues after celecoxib treatment. **B** Celecoxib treatment reduced the average volume of the xenograft tumors formed by FADS1-OE cells.

**Supplementary datasetⅠ**The microarray original data.

**Supplementary dataset Ⅱ** The information of the PPI network

**Table. SⅠA** The sequences of FADS1- shRNAs

**Table. SⅠB** The sequences of the qRT-PCR primers used in this study.

**Table. SⅡ** Relationship between clinicopathological factors and FADS1 expression in 110 LSCC patients.

**Table SⅢ** The details of annotated genes
