# Supplementary figures and images for: FADS1 promotes the progression of laryngeal squamous cell carcinoma through activating AKT/mTOR signaling
Source: Cell Death Dis. 2020 Apr 24;11(4):272. doi: 10.1038/s41419-020-2457-5 (PMC7181692; doi:10.1038/s41419-020-2457-5)

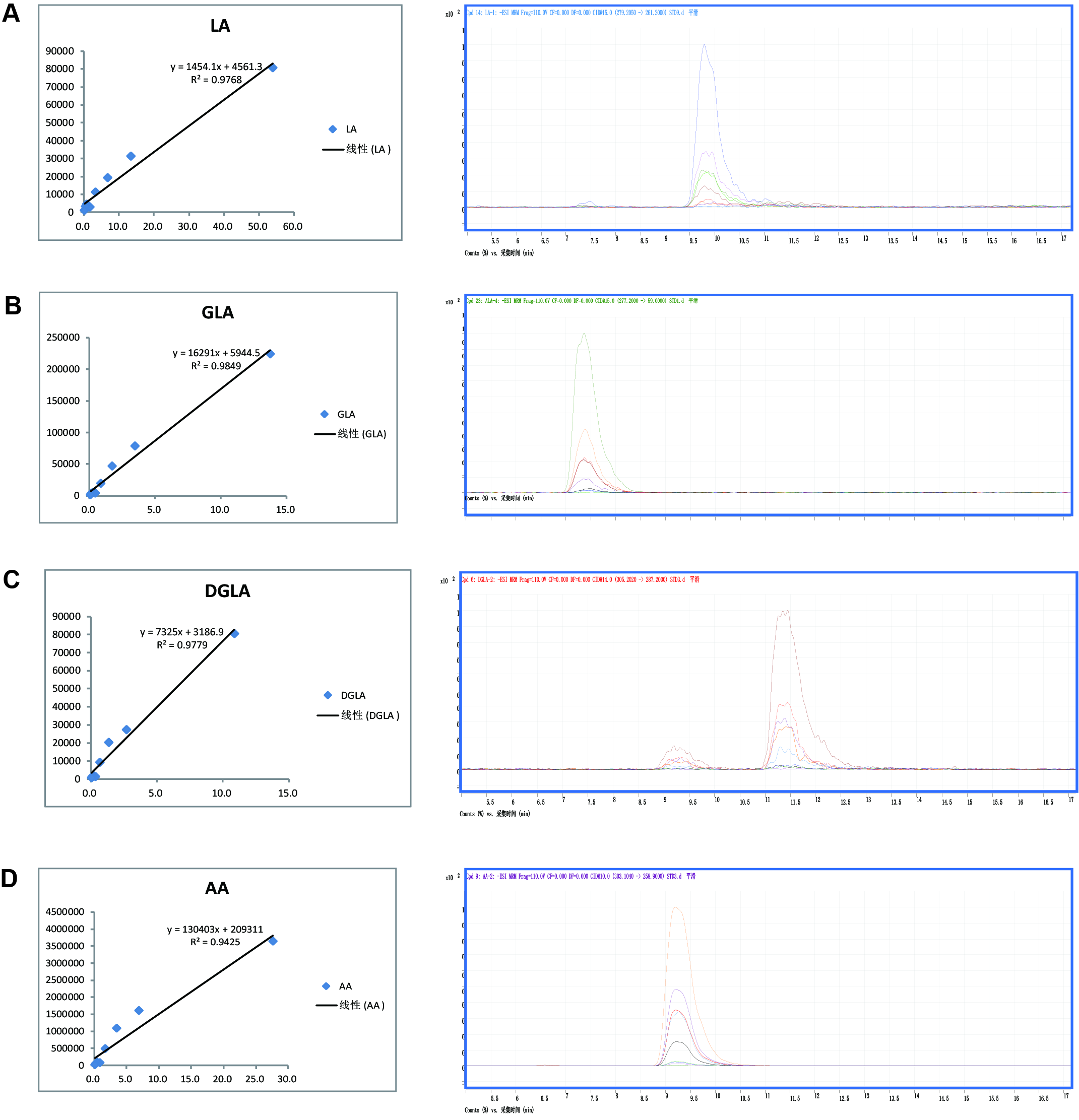

Supplement: Supplementary file 2 — Supplementary Fig. 1 [file 41419_2020_2457_MOESM2_ESM.tif]

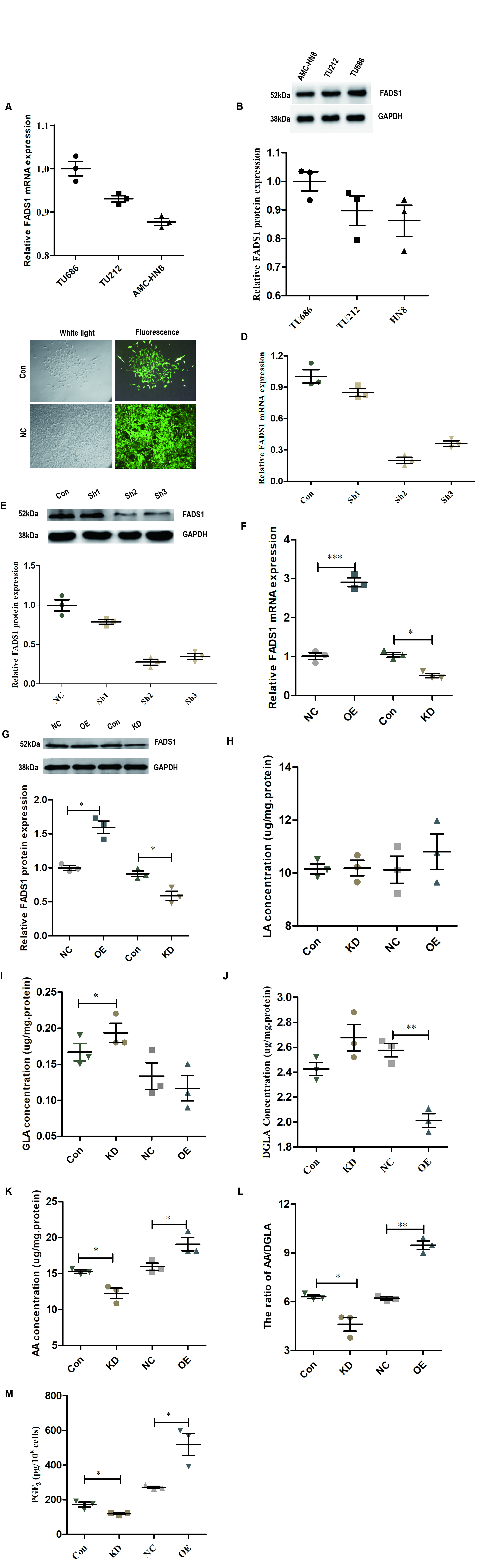

Supplement: Supplementary file 3 — Supplementary Fig. 2 [file 41419_2020_2457_MOESM3_ESM.tif]

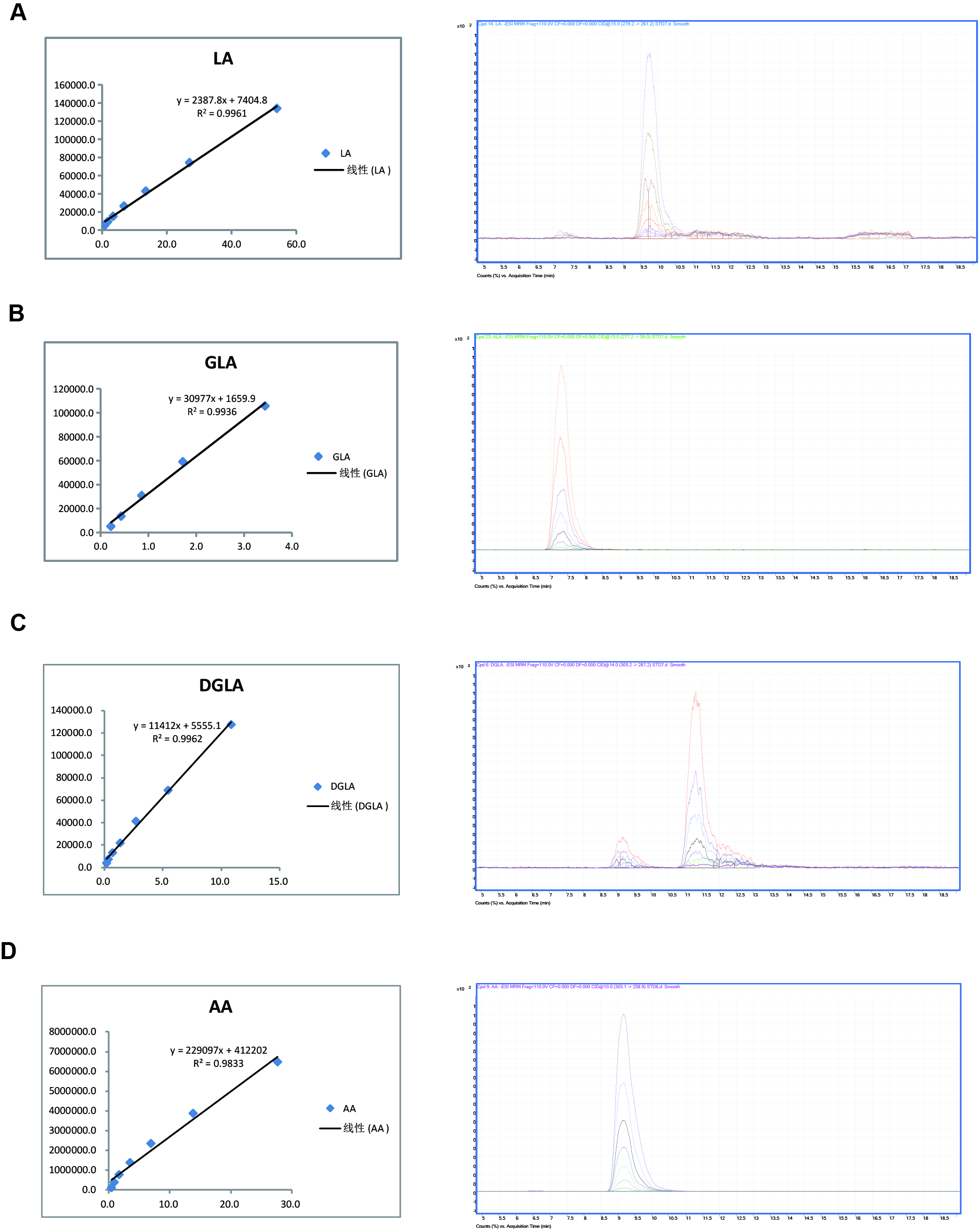

Supplement: Supplementary file 4 — Supplementary Fig. 3 [file 41419_2020_2457_MOESM4_ESM.tif]

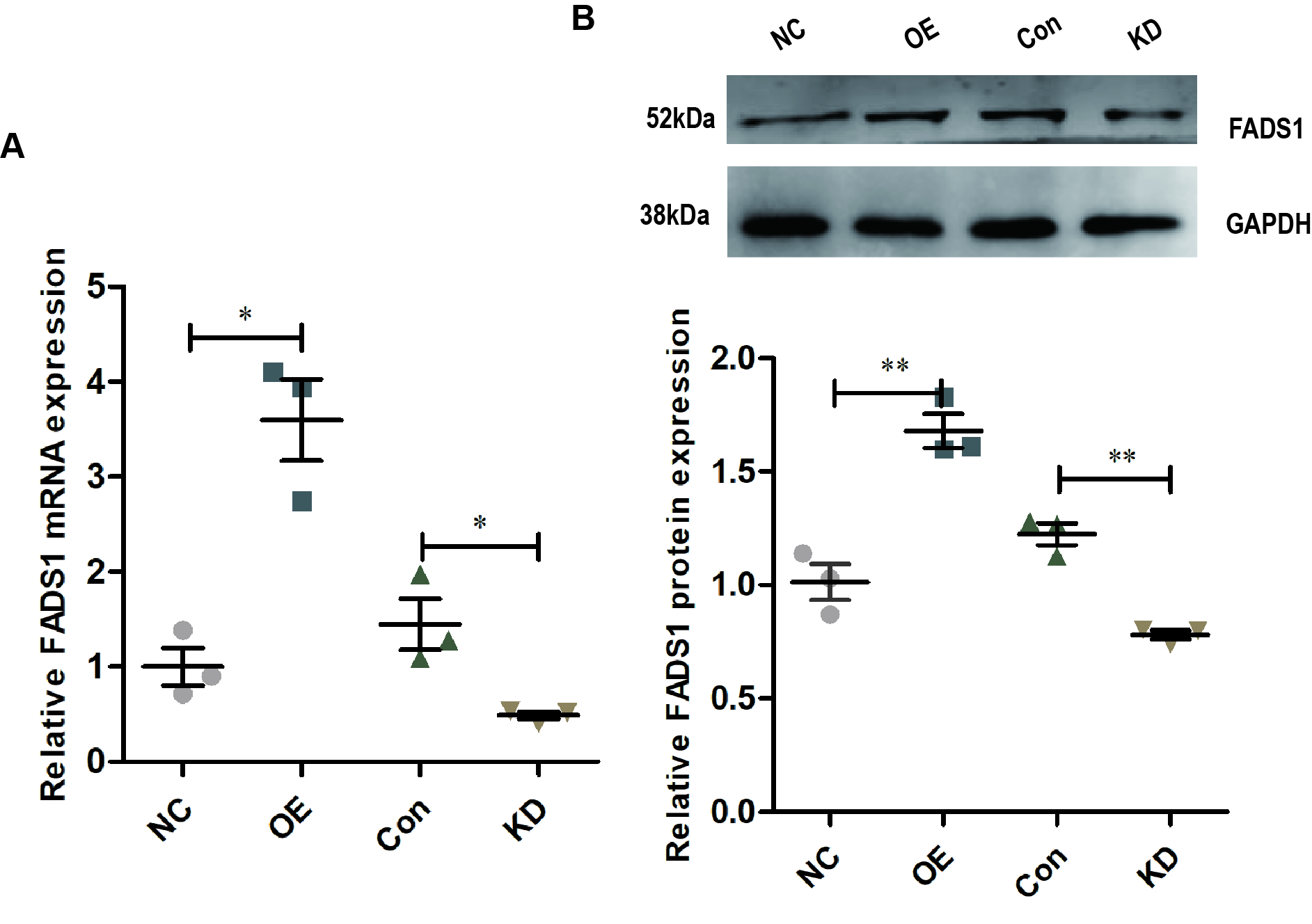

Supplement: Supplementary file 5 — Supplementary Fig. 4 [file 41419_2020_2457_MOESM5_ESM.tif]

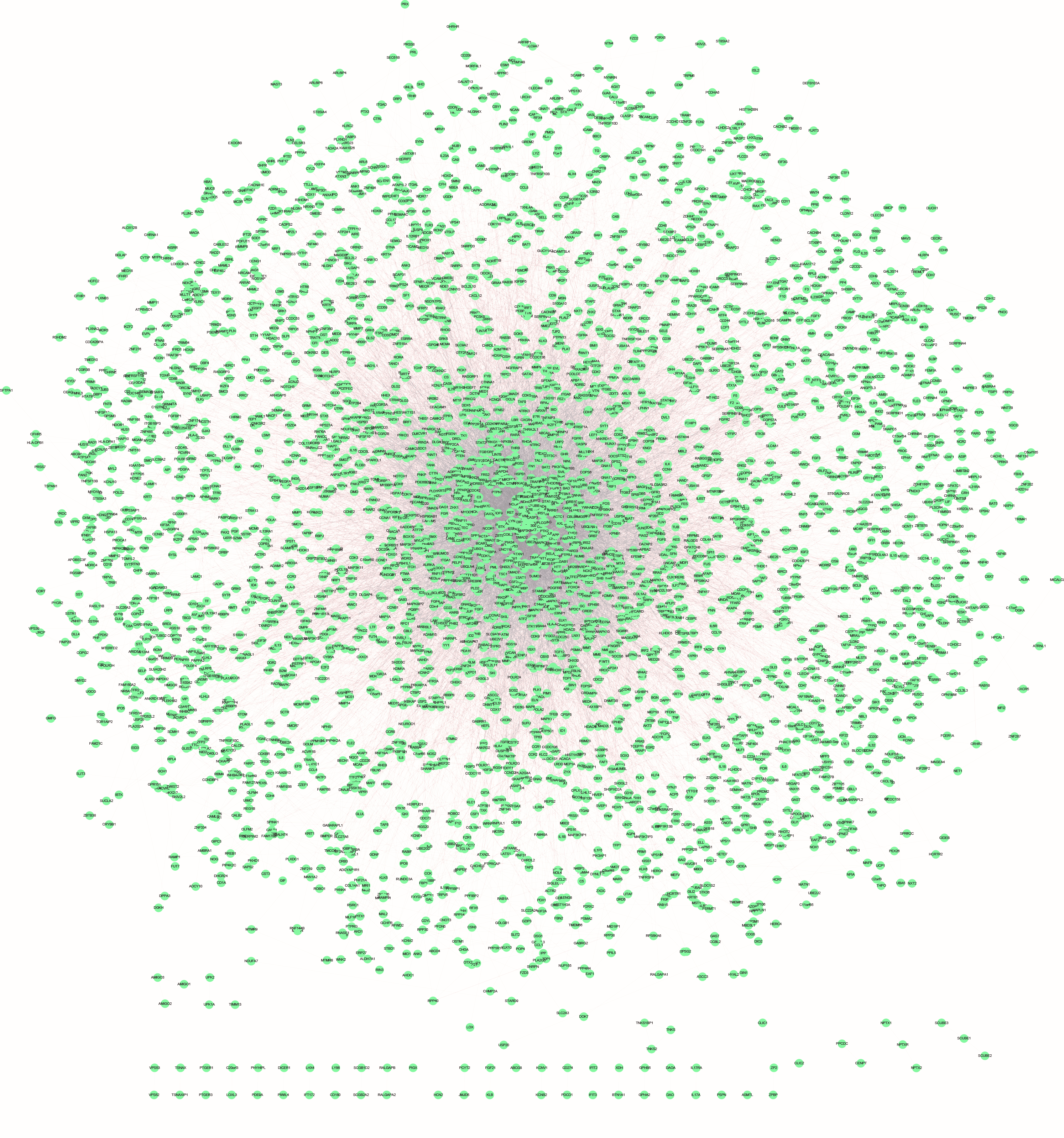

Supplement: Supplementary file 6 — Supplementary Fig. 5 [file 41419_2020_2457_MOESM6_ESM.tif]

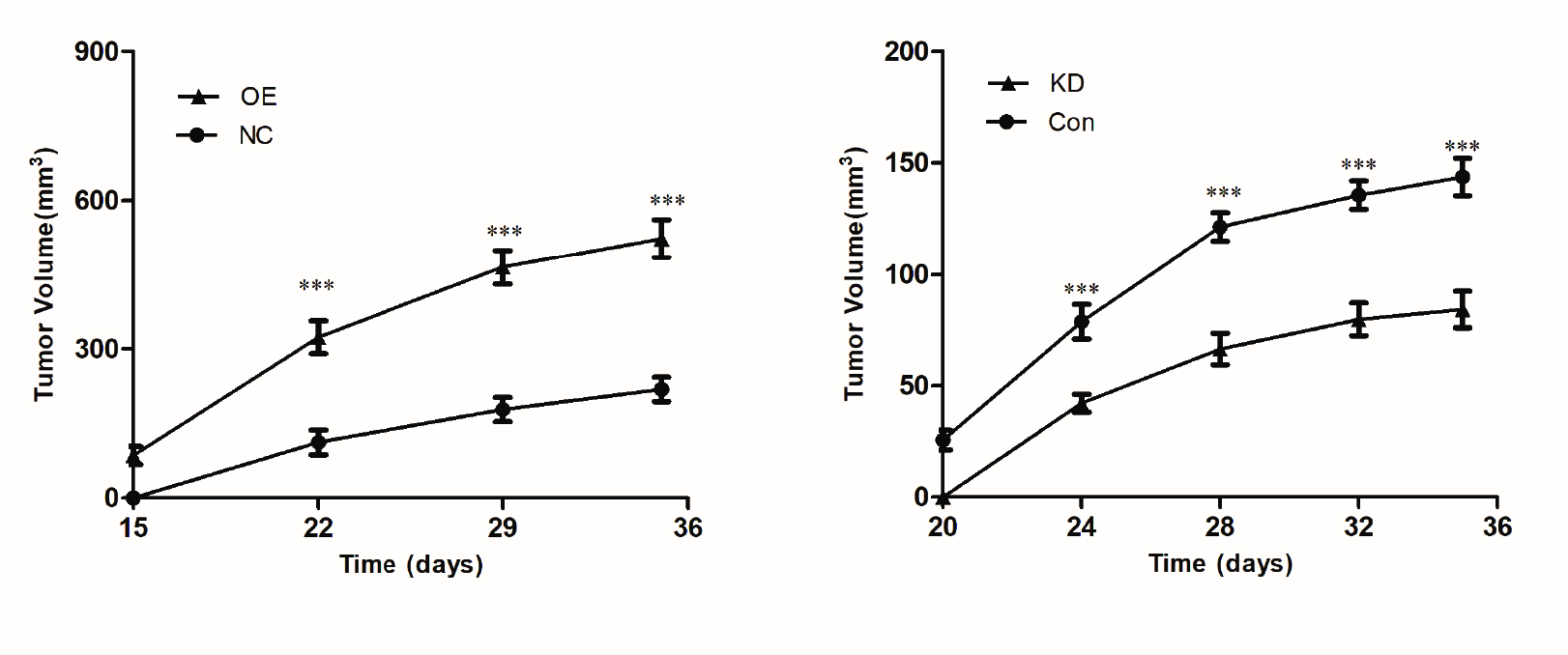

Supplement: Supplementary file 7 — Supplementary Fig. 6 [file 41419_2020_2457_MOESM7_ESM.tif]

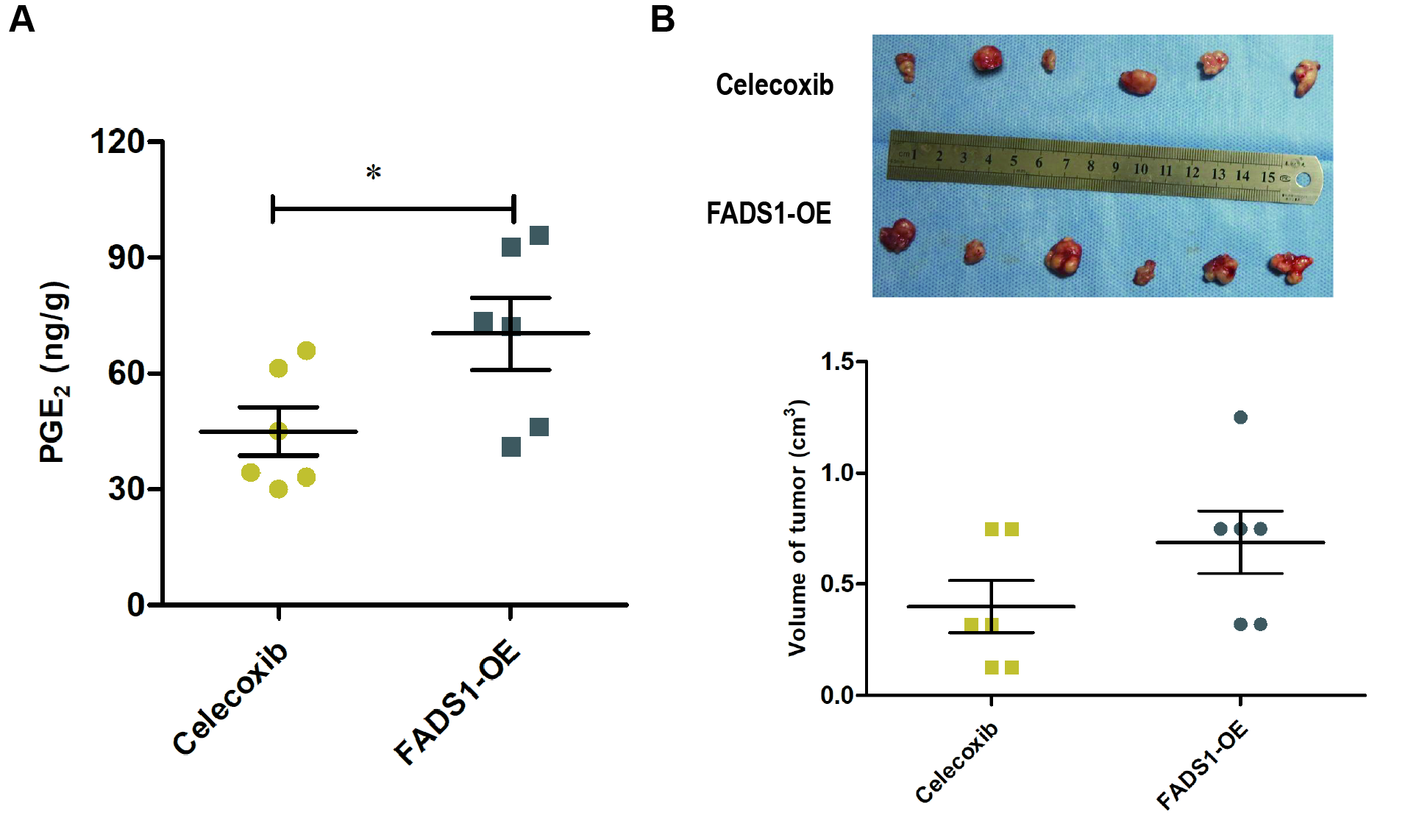

Supplement: Supplementary file 8 — Supplementary Fig. 7 [file 41419_2020_2457_MOESM8_ESM.tif]
