# Supplementary material for: FADS1 promotes the progression of laryngeal squamous cell carcinoma through activating AKT/mTOR signaling
Source: Cell Death Dis. 2020 Apr 24;11(4):272. doi: 10.1038/s41419-020-2457-5 (PMC7181692; doi:10.1038/s41419-020-2457-5)
Supplement: Supplementary file 9 — Table SI [file 41419_2020_2457_MOESM9_ESM.docx]

**Table. SⅠA** The sequences of FADS1- shRNAs

| Lentivirus name | Sequence |
| --- | --- |
| FADS1-ShRNA1: | CCGGGGTGACACAGATGAACCATATCTCGAGATATGGTTCATCTGTGTCACCTTTTT |
| FADS1-ShRNA2: | CCGGGCGTATTCACCACAGGAATCACTCGAGTGATTCCTGTGGTGAATACGCTTTTT |
| FADS1-ShRNA3: | CCGGGCAAAGACCCAGACATCAACACTCGAGTGTTGATGTCTGGGTCTTTGCTTTTT |

**Table. SⅠB** The sequences of the qRT-PCR primers used in this study.

| Gene Forward primer Reverse primer |
| --- |
| FADS1 CTACCCCGCGCTACTTCAC CGGTCGATCACTAGCCACC  BAX TGGCAGCTGACATGTTTTCTGAC TCACCCAACCACCCTGGTCTT  PTEN ACCAGGACCAGAGGAAACCT GCTAGCCTCTGGATTTGACG  mTOR CGCTGTCATCCCTTTATCG ATGCTCAAACACCTCCACC  HOXD4 CCCTCCGTGCGAGGAGTAT GAAAGGCTGCTCACCGAAGT  AKT1 TGTCTGTCACCAGCTATCTG AAATTTAAACCTTGCTCCTC  β-ACTIN AGCGAGCATCCCCCAAAGTT AGGGGCCATCCACAGTCTTC |
