# Supplementary material for: FADS1 promotes the progression of laryngeal squamous cell carcinoma through activating AKT/mTOR signaling
Source: Cell Death Dis. 2020 Apr 24;11(4):272. doi: 10.1038/s41419-020-2457-5 (PMC7181692; doi:10.1038/s41419-020-2457-5)
Supplement: Supplementary file 10 — Table SII [file 41419_2020_2457_MOESM10_ESM.docx]

Table. SⅡ Relationship between clinicopathological factors and FADS1 expression in 110 LSCC patients.

|  | FADS1 expression | | |
| --- | --- | --- | --- |
| Clinic pathological characteristics | FADS1≤4 | FADS1>4 | p-value |
| n=110 | 65(59.09) | 45(40.91） |  |
| Age |  |  | 0.814 |
| Age<60 | 39 | 28 |  |
| Age≥60 | 26 | 17 |  |
| Gender |  |  | 0.698 |
| Male | 47 | 31 |  |
| Female | 18 | 14 |  |
| T classification |  |  | 0.009 |
| T1-2 | 41 | 17 |  |
| T3-4 | 24 | 28 |  |
| Lymph |  |  | 0.058 |
| N0 | 36 | 16 |  |
| N1-3 | 29 | 29 |  |
| Pathology differentiation |  |  | 0.235 |
| Low | 15 | 15 |  |
| High-Mild | 50 | 30 |  |
| Smoking |  |  | 0.822 |
| Yes | 60 | 41 |  |
| No | 5 | 4 |  |
| Drinking |  |  | 0.672 |
| Yes | 43 | 28 |  |
| No | 22 | 17 |  |
| Primary location |  |  | 0.26 |
| Supraglottic | 41 | 33 |  |
| Glottis | 24 | 12 |  |
| Clinical stage |  |  | 0.014 |
| I-II | 24 | 7 |  |
| III-IV | 41 | 38 |  |
| Recurrence |  |  | 0.598 |
| Yes | 45 | 29 |  |
| No | 20 | 16 |  |
