# Supplementary material for: FADS1 promotes the progression of laryngeal squamous cell carcinoma through activating AKT/mTOR signaling
Source: Cell Death Dis. 2020 Apr 24;11(4):272. doi: 10.1038/s41419-020-2457-5 (PMC7181692; doi:10.1038/s41419-020-2457-5)
Supplement: Supplementary file 11 — Table SIII [file 41419_2020_2457_MOESM11_ESM.docx]

**Table SⅢ** The details of annotated genes

| Pathway ID | Definition | Fisher-Pvalue | Genes |
| --- | --- | --- | --- |
| hsa03320 | PPAR signaling pathway - Homo sapiens (human) | 0.000978295 | ACOX2//ACSL5//FABP3//PLIN2//PPARA//PPARG//  SCD5//SLC27A6//SORBS1 |
| hsa05200 | Pathways in cancer - Homo sapiens (human) | 0.001965768 | AKT1//BIRC3//BMP4//CCNA1//CCNE2//CDH1// E2F3//FGF17//FGFR1//FGFR3//FRAT1//GNG2// HDAC2//IFNAR2//IL6ST//IL7//IL7R//ITGA2//KLK3// MAPK10//MAPK9//MTOR//NOTCH4//PGF// PPARG//PTEN//PTGER3//PTGS2//RASGRP3//  RPS6KB1//RASSF5//WNT4 |
| hsa04015 | Rap1 signaling pathway - Homo sapiens (human) | 0.002783601 | AKT1//APBB1IP//CDH1//CNR1//CTNND1//FGF17//  FGFR1//FGFR3//ITGB2//MAGI1//P2RY1//PFN4// PGF//RAPGEF6//RASGRP3//RASSF5 |
| hsa04920 | Adipocytokine signaling pathway –Homo sapiens (human) | 0.01117174 | ACSL5//AKT1//MAPK10//MAPK9//PPARA//  PPARGC1A//PTPN11 |
| hsa04950 | Maturity onset diabetes of the young –  Homo sapiens (human) | 0.01279256 | HNF4G//NEUROD1//NR5A2//PAX6 |
| hsa04931 | Insulin resistance - Homo sapiens (human) | 0.01399657 | AKT1//MAPK10//MAPK9//PPARA//PPARGC1A//  PTEN//PTPN11//RPS6KA6//SLC27A6 |
| hsa04068 | FoxO signaling pathway - Homo sapiens (human) | 0.01942777 | AKT1//CCNB3//FOXO4//IL7R//MAPK10//MAPK9//  PTEN//RAG1//S1PR1//TNFSF10 |
| hsa00512 | Mucin type O-glycan biosynthesis –  Homo sapiens (human) | 0.02343834 | GALNT13//GALNT4//GALNT8//GCNT1 |
| hsa04550 | Signaling pathways regulating pluripotency  of stem cells - Homo sapiens (human) | 0.0267391 | ACVR1C//AKT1//BMP4//FGFR1//FGFR3//IL6ST//  PAX6//REST//SKIL//WNT4 |
| hsa04630 | JAK-STAT signaling pathway –  Homo sapiens (human) | 0.030137 | AKT1//CSF2RA//IFNAR2//IL20RB//IL6ST//IL7//  IL7R//PIAS4//PRLR//PTPN11//SOCS6 |
| hsa04514 | Cell adhesion molecules (CAMs) –  Homo sapiens (human) | 0.03304543 | CADM1//CDH1//CDH15//ITGB2//NCAM1//  NRXN1//NRXN3//PTPRC//PTPRM//PVR |
| hsa04014 | Ras signaling pathway - Homo sapiens (human) | 0.03793414 | AKT1//FGF17//FGFR1//FGFR3//FOXO4//GNG2// MAPK10//MAPK9//NTRK2//PGF//PTPN11//RASAL  //RASGRP3//RASSF5 |
| hsa05340 | Primary immunodeficiency –  Homo sapiens (human) | 0.04170858 | BTK//IL7R//PTPRC//RAG1 |
| hsa05218 | Melanoma - Homo sapiens (human) | 0.04299265 | AKT1//CDH1//E2F3//FGF17//FGFR1//PTEN |
| hsa05222 | Small cell lung cancer - Homo sapiens (human) | 0.04802343 | AKT1//BIRC3//CCNE2//E2F3//ITGA2//PTEN//  PTGS2 |
| hsa04151 | PI3K-Akt signaling pathway – Homo sapiens (human) | 0.04896898 | AKT1//CCNE2//FGF17//FGFR1//FGFR3//GNG2// IFNAR2//IL7//IL7R//ITGA1//ITGA2//MAGI1// MTCP1//NTRK2//PGF//PHLPP2//PRLR//PTEN//  TSC2 |
